# Enhancement of rat lymphatic lipid transport by glucose or amino acids ingestion

**DOI:** 10.14814/phy2.14079

**Published:** 2019-04-23

**Authors:** Hiroshi Hayashi

**Affiliations:** ^1^ Department of Internal Medicine Tokyo Ariake University of Medical and Health Sciences Koto‐ku Tokyo Japan

**Keywords:** Amino acids, glucose, intestine, lipid, lymph

## Abstract

To elucidate the effect of simultaneously fed carbohydrate or protein on lipid absorption, lymphatic lipid transports in the rat intestine were observed with or without simultaneous feeding of glucose or amino acids. A lipid emulsion containing 40 *μ*mol/h (35.4 mg/h) of triolein, 2.74 *μ*mol/h (1.06 mg/h) of cholesterol, 7.8 *μ*mol/h (6.08 mg/h) of egg phosphatidylcholine without any additive (the Control group), with 560 mmol/h (300 mg/h) of glucose (the Glucose group), or with 400 mmol/h (150 mg/h) of amino acids (the Amino Acids group), was infused intraduodenally at 3 mL/h for 8 h into mesenteric lymph‐fistula rats. The amounts of triglyceride transported in lymph for 8 h were 185 ± 12 (mean ± SE) mg in the Amino Acids group (*n* = 4), 175 ± 3 mg in the Glucose group (*n* = 5), and 147 ± 7 mg in the Control group (*n* = 4), respectively, with a statistically significant difference (*P* < 0.05) among the groups. The amounts of cholesterol transported in lymph for 8 h of the Amino Acid group and the Glucose group seemed to be larger than that of the Control group. The amount of phosphatidylcholine transported in lymph for 8 h were 16.4 ± 1.0 mg in the Amino Acids group, 15.7 ± 0.4 mg in the Glucose group, and 12.4 ± 0.3 mg in the Control group, respectively, with a statistically significant difference (*P* < 0.01) among the groups. Simultaneous glucose or amino acids feeding enhanced lymphatic lipid transport in the rat intestine during lipid feeding.

## Introduction

Hyperlipidemia is recognized as a major risk factor for atherosclerotic cardiovascular diseases (Matsuura et al. 2019). Plasma lipids are derived not only from the de novo synthesis in the body but also from the diet. When the plasma lipid metabolism is considered, dietary lipids must always be taken into account. Dietary lipid absorption usually occurs with absorption of other nutrients because lipid is contained in foods fed as a meal. As nutrients contained in the food are suspected to interact with each other during their intestinal absorption, researchers have sought the relationship among the nutrients in the intestinal absorption. For example, the composition of nutrients providing optimal absorption rates in mini pigs was suggested (Weber and Ehrlein 1999). The vegetable oil and starch were shown to negatively affect cholesterol (Ch) absorption in a fish, gilthead sea bream juveniles (Castro et al. 2016).

The studies about the effect of other nutrients on intestinal lipid absorption in the mammals are limited so far. Meyer et al. (1976) reported that protein stabilizes lipid emulsions leading to facilitation of fat absorption in the rats. The Tso's laboratory observed the effect of amino acids on intestinal lipid absorption using mesenteric lymph fistula rats. They showed that enterally administered l‐glutamine promoted lymphatic lipid transport in a dose‐dependent manner (Schwimmer et al. 2002) and that, on the other hand, l‐glutamate inhibited lymphatic lipid transport using the same rat model (Kohan et al. 2016). Dietary carbohydrates or glucose were shown to enhance intestinal lipid absorption and chylomicron secretion in humans (Harbis et al. 2001; Morgantini et al. 2014). This was concluded by the measurement of apolipoprotein B‐48 (apoB48), triglyceride (TG)‐rich lipoproteins in postprandial plasma because apoB48 is exclusively produced by the enterocytes in humans.

After hydrolysis by pancreas enzymes, dietary lipids are absorbed into the enterocytes, re‐esterified, packed in the chylomicrons, and finally transported into the intestinal lymph. Therefore, to observe the lipid transport in the intestinal lymph is the comprehensive measurement of intestinal lipid absorption. The mesenteric lymph fistula rat is a very suitable animal model for this purpose (Tso 1985).

The objective of this study is to directly analyze the effect of simultaneously fed glucose or amino acids on intestinal lipid absorption using the mesenteric lymph fistula rats to seek the interactions among the nutrients in the food on their absorption in the intestine.

## Materials and Methods

### Animals

Male Sprague Dawley rats (body weight, 300–350 g) were purchased from Sankyo Lab Service (Tokyo, Japan). All animal procedures were performed in accordance with approval by the Animal Experiments Committee of the Tokyo Ariake University of Medical and Health Sciences.

### Lymph cannulation and duodenum intubation

The rats were fasted overnight. The rats were anesthetized with isoflurane and buprenorphine was given to alleviate pain associated with surgery. The main mesenteric lymph duct was cannulated with clear vinyl tubing (0.8 mm OD) according to the method of Bollman et al. (1948). Silicone tubing (2.2 mm OD) was introduced 2 cm down the duodenum through the fundus of the stomach, and the fundal incision was closed with a purse‐string suture. Postoperatively, the rats were allowed to recover in restraint cages and intraduodenally infused with saline (145 mmol/L NaCl, 4 mmol/L KCl) at 3 mL/h.

### Lipid emulsions

Three kinds of lipid emulsion were prepared. The lipid emulsion for the Control group containing 40 *μ*mol/h (35.4 mg/h) of triolein, 2.74 *μ*mol/h (1.06 mg/h) of Ch, 7.8 *μ*mol/h (6.08 mg/h) of egg phosphatidylcholine (PC), and 57 *μ*mol/h of taurocholate, emulsified in phosphate‐buffered saline (pH 6.4) was made as previously described (Hayashi et al. 2002). For the Glucose group, a quantity of 560 mmol/h (300 mg/h) of glucose was added to the lipid emulsion of the Control group. For the Amino Acids group, 400 mmol/h (150 mg/h), which was calculated according to the amino acids composition described below, of amino acids derived from a medical amino acid injection (Amizet^®^B, Terumo, Tokyo, Japan), which consists of 8.5% l‐isoleucine, 13.5% l‐leucine, 8% l‐lysine, 3.9% l‐methionine, 7.7% l‐phenylalanine, 4.8% l‐threonine, 1.6% l‐tryptophan, 9% l‐valine, 1% l‐cysteine, 0.5% l‐tyrosine, 11.1% l‐arginine, 4.7% l‐histidine, 8.6% l‐alanine, 0.5% l‐aspartate, 0.5% l‐ glutamate, 5.5% glycine, 6.4% l‐proline, and 4.2% l‐serine, was added to the lipid emulsion of the Control group.

### Experimental protocols

On the day after surgery, the saline infusion was replaced by a lipid emulsion. The rats were divided into the three groups, that is, the Control group, the Glucose group, and the Amino Acids Group. Each group was infused with the designated lipid emulsion described above at 3 mL/h for 8 h. Lymph was collected before and hourly during the lipid infusion. At the end of the infusion period, the rats were anesthetized with isoflurane and killed by exsanguination. Concentrations of glucose, TG, Ch, and PC in the lymph were measured enzymatically.

### Statistical analysis

A repeated measures analysis of the variance test was used to determine whether differences existed among the groups. The Student's *t*‐test for independent means was used where appropriate. Differences with a *P* < 0.05 were considered as significant. All statistical analyses were performed with the SPSS statistical package (Chicago, IL).

## Results

### Lymph flow

**Figure 1:**
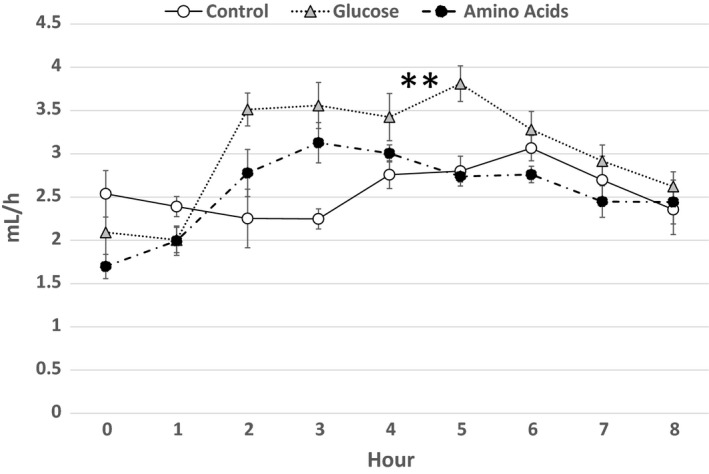
Lymph flow. Lymph flow rates (mL/h) of the Control group (*n* = 4), the Glucose group (*n* = 5), and the Amino Acids group (*n* = 4) are depicted as an open circle, a gray triangle, and a closed circle, respectively. Each mark represents mean ± SE. Lymph flow rates increased in each group after the lipid infusion with the significantly (*P* < 0.01) larger increase in the Glucose group than in the other two groups denoted by double asterisk.

Lymph flow rate 1 h before the lipid infusion was 2.54 ± 0.27 (mean ± SE) mL/h in the Control group (*n* = 4), 2.09 ± 0.42 mL/h in the Glucose group (*n* = 5), and 1.70 ± 0.14 mL/h in the Amino Acids group (*n* = 4), respectively, with no significant difference (Fig. 1). Lymph flow rates increased in each group after the lipid infusion with the significantly (*P* < 0.01) larger increase in the Glucose group than in the other two groups.

### Glucose concentration in lymph

**Figure 2:**
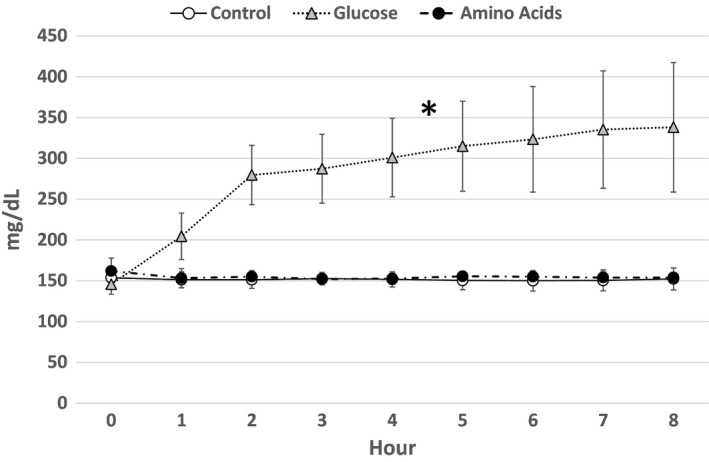
Glucose concentration in lymph. Glucose concentrations (mg/dL) in the lymph of each group are shown. The symbols are as same as Figure 1. Because the data of the Control group and the Amino Acids group are almost same during the lipid infusion, both symbols are depicted as overlapped. While the glucose concentrations of the lymph in the Control and Amino Acids groups did not change after the lipid infusion, the glucose concentrations of the lymph in the Glucose group increased significantly (*P* < 0.05) after the lipid infusion denoted by single asterisk.

Glucose concentration in the lymph 1 h before the lipid infusion was 154.8 ± 8.9 mg/dL in the Control group, 145.8 ± 12.4 mg/dL in the Glucose group, and 162.0 ± 15.7 mg/dL in the Amino Acids group, respectively, with no significant difference (Fig. 2). While the glucose concentrations of the lymph in the Control and Amino Acids groups did not change after the lipid infusion, the glucose concentrations of the lymph in the Glucose group increased significantly after the lipid infusion (*P* < 0.05). The lymphatic glucose concentrations in 8 h were 152.3 ± 13.5 mg/dL in the Control group, 338.2 ± 79.3 mg/dL in the Glucose group, and 154.0 ± 6.3 mg/dL in the Amino Acids group, respectively.

### TG transport in lymph

**Figure 3:**
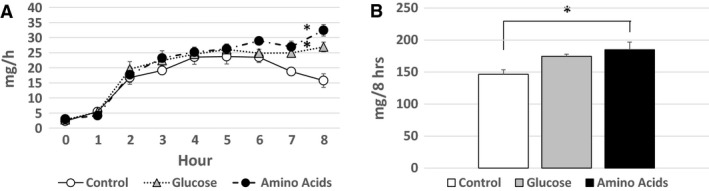
TG transport in lymph. The amounts (mg/h) of TG transported hourly in the lymph (A) and the total amounts (mg) of TG transported in the lymph during the 8 h’ lipid infusion (B) are shown. The symbols are as same as Figure 1. The amounts of TG transported hourly in the lymph increased in each group after the lipid infusion with the significantly (*P* < 0.05) larger increase in the Glucose and Amino Acids groups than in the Control group as denoted by single asterisk (A). There is a significant (*P* < 0.05) difference of the total amounts of TG transported in the lymph during the 8 h’ lipid infusion between the Control group and Amino Acids group as denoted by single asterisk (B).

The amount of TG transported in the lymph 1 h before the lipid infusion was 2.33 ± 0.14 mg/h in the Control group, 2.77 ± 0.30 mg/h in the Glucose group, and 3.01 ± 0.24 mg/h in the Amino Acids group, respectively, with no significant difference. The amounts of TG transported hourly in the lymph increased in each group after the lipid infusion with the significantly (*P* < 0.05) larger increase in the Glucose and Amino Acids groups than in the Control group as shown in Figure 3A. The total amounts of TG transported in lymph during the 8 h’ lipid infusion were 146.6 ± 7.0 mg in the Control group, 174.6 ± 3.3 mg in the Glucose group, and 184.9 ± 11.9 mg in the Amino Acids group, respectively, with a significant (*P* < 0.05) difference between the Control group and Amino Acids group (Fig. 3B).

### Ch transport in lymph

**Figure 4:**
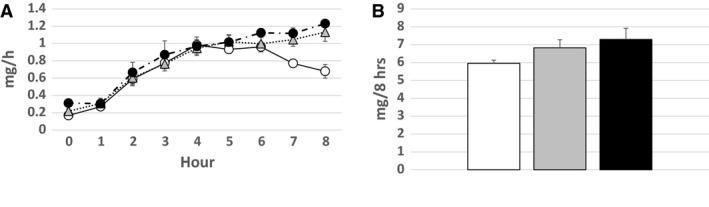
Ch transport in lymph. The amounts (mg/h) of Ch transported hourly in the lymph (A) and the total amounts (mg) of Ch transported in the lymph during the 8 h’ lipid infusion (B) are shown. The symbols are as same as Figure 3. The amount of Ch transported hourly in the lymph increased in each group after the lipid infusion. While the Ch transported hourly in the lymph in the Glucose and Amino Acids groups seemed to be more transported than that in the Control group at the later period of the lipid infusion (A), the total amount of Ch transported in the lymph during the 8 h’ lipid infusion was not significantly different among the three groups (B).

The amount of Ch transported in the lymph 1 h before the lipid infusion was 0.17 ± 0.02 mg/h in the Control group, 0.22 ± 0.03 mg/h in the Glucose group, and 0.31 ± 0.04 mg/h in the Amino Acids group, respectively, with no significant difference. The amount of Ch transported hourly in the lymph increased in each group after the lipid infusion. While the Ch in the Glucose and Amino Acids groups seemed to be transported more than that in the Control group at the later period of the lipid infusion (Fig. 4A), the total amount of Ch transported in the lymph during the 8 h’ lipid infusion was not significantly different among the three groups (Fig. 4B).

### PC transport in lymph

**Figure 5:**
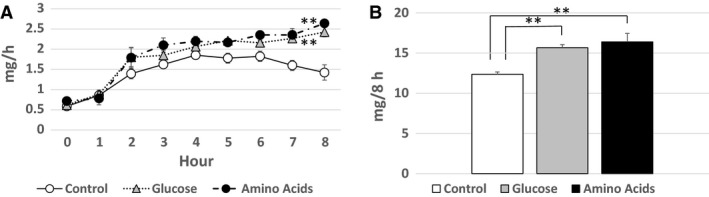
PC transport in lymph. The amounts (mg/h) of PC transported hourly in the lymph (A) and the total amounts (mg) of PC transported in the lymph during the 8 h’ lipid infusion (B) are shown. The symbols are as same as in Figure 3. The amounts of PC transported hourly in the lymph increased in each group after the lipid infusion with the significantly (*P* < 0.01) larger increase in the Glucose and Amino Acids groups than in the Control group as denoted by double asterisk (A). There is a significant (*P* < 0.01) difference of the total amounts of PC transported in the lymph during the 8 h’ lipid infusion both between the Control group and the Glucose group and between the Control group and Amino Acids group as denoted by double asterisk (B).

The amount of PC transported in the lymph 1 h before the lipid infusion was 0.58 ± 0.04 mg/h in the Control group, 0.62 ± 0.06 mg/h in the Glucose group, and 0.72 ± 0.08 mg/h in the Amino Acids group, respectively, with no significant difference. The amounts of PC transported hourly in the lymph increased in each group after the lipid infusion with the significantly (*P* < 0.01) larger increase in the Glucose and Amino Acids groups than in the Control group as shown in Figure 5A. The total amounts of PC transported in lymph during the 8 h’ lipid infusion were 12.4 ± 0.3 mg in the Control group, 15.7 ± 0.4 mg in the Glucose group, and 16.4 ± 1.0 mg in the Amino Acids group, respectively, with a significant (*P* < 0.01) difference both between the Control group and the Glucose group and between the Control group and the Amino Acids group (Fig. 5B).

## Discussion

The transport of lipid, especially of TG and PC, in the lymph was enhanced by the simultaneous infusion of glucose or amino acids in this study. The increment in lipid transport was more prominent in the later period of 8 h’ lipid infusion in either cases of adding glucose or amino acids. Comparing the two nutrients, the amino acids seemed to affect the lymphatic lipid transport more than the glucose in this experimental condition. The glucose concentration in lymph of the Glucose group increased twice more than that of the other two groups. While the glucose in the peripheral portal vein was supposed to infiltrate into the lymph duct, it is possible that the glucose was directly secreted into the lymph from the enterocytes after the absorption. The direct secretion of glucose to the lymph from the gut has been reported by other investigators (Fernández‐López et al. 1992). The high concentration of glucose in the lymph of the Glucose group seemed to cause the increased osmotic pressure in the lymph resulting in the increased lymph flow rate observed in the Glucose group compared to the other two groups. Because the amount of lipid transported in the lymph was calculated as the mass, but not the concentration, in this study, the difference in lymph flow rates among the three experimental groups does not affect the interpretation of the results of lymphatic lipid net transport.

Although it is of interest to consider how the other nutrients enhanced the lymphatic lipid transport in this study, the experiments to find out the mechanism about the effect of other nutrients on lipid transport was not performed in this experiment. The experiments whether the enhancement of lipid transport is similarly observed in other experimental conditions of adding other nutrients must also be necessary to find the mechanism. Therefore, the discussion on mechanism is limited to a speculation referring to the literature.

As already mentioned, dietary carbohydrates or glucose were shown to enhance intestinal lipid absorption and chylomicron secretion in humans (Harbis et al. 2001; Morgantini et al. 2014). Dash et al. (2014) showed that glucagon‐like peptide‐2 (GLP‐2) facilitates this process. GLP‐2 is one of the intestinotropic peptides that is produced in enteroendocrine L cells of the small and large intestine and secreted in a nutrient‐dependent manner. GLP‐2 is encoded, together with glucagon and glucagon‐like peptide‐1 (GLP‐1), by a proglucagon gene (Drucker 2001). Administration of GLP‐2 to men caused the release of chylomicrons that comprise previously synthesized and stored apoB48 and lipids (Dash et al. 2014). In the Syrian golden hamster, an intravenous infusion of GLP‐2 resulted in a marked increase in postprandial apoB48 and TG levels in TG‐rich lipoprotein fraction, whereas GLP‐1 infusion decreased lipid absorption and levels of TG and apoB48 in TG‐rich lipoprotein (Hein et al. 2013). If GLP‐2 is also involved in the enhancement of lymphatic lipid transport by glucose or amino acids shown in this study, measurements of GLP‐2 as same as GLP‐1 may be crucial. As intestinal lymph is reported to contain high concentrations of postprandial GLP‐1 (Lu et al. 2007), lymph must be a suitable sample to measure both GLP‐1 and GLP‐2.

In the studies of humans (Harbis et al. 2001; Dash et al. 2014; Morgantini et al. 2014) and Syrian golden hamsters (Hein et al. 2013), ingestion of glucose with lipid and infusion of GLP‐2, respectively, caused postprandial increase of TG and apoB48 in plasma. We showed that lipid ingestion without other nutrients results in increasing the size, but not the number, of chylomicrons in lymph, which is concluded by the observation that the mass of apoB transported in lymph does not change during lipid ingestion (Hayashi et al. 1990). Therefore, it would be interesting to measure apoB48 in lymph in this study with regard to the mechanism of the enhancement of lymphatic lipid transport by other nutrients.

As already mentioned, lymphatic lipid transport was enhanced or inhibited by co‐administration of l‐glutamine (Schwimmer et al. 2002) or _L_‐glutamate (Kohan et al. 2016), respectively. In this study, a mixed preparation of amino acids for a medical use of intravenous injections was tested and it promoted lymphatic lipid transport by the simultaneous ingestion. Because each protein consists of a different percentage of amino acids, there may be several cases in the effect of simultaneously ingested protein on lymphatic lipid transport depending on each protein. If protein ingestion generally enhances lymphatic lipid transport as shown in this study, it is possible that GLP‐2 is involved in this process because amino acids or protein ingestion also facilitates GLP‐2 secretion (Kieffer and Habener 1999).

Another possibility of the mechanism of the enhancement of the lymphatic lipid transport by glucose or amino acids shown in this study is that the extra calories infused by glucose or amino acids somehow increased the lymphatic lipid transport. While lipid infusion in the Control group fed rats with 400 calories/h, 1200 calories/h were more added in the Glucose group and 600 calories/h were more added in the Amino Acids group, respectively, in this study. It is possible that the extra calories infused into the rats affected the lymphatic lipid transport by some mechanism.

In conclusion, this study showed that the simultaneous glucose or amino acids feeding enhanced lymphatic lipid transport in the rat intestine during lipid feeding and further investigations deserve to elucidate the mechanism of this finding.

## Conflicts of Interest

The author has no conflicts of interest to report.
